# Supplementary material for: Uniform bacterial genetic diversity along the gut
Source: Nat Commun. 2026 Mar 17;17:4100. doi: 10.1038/s41467-026-70705-8 (PMC13144417; doi:10.1038/s41467-026-70705-8)
Supplement: Supplementary file 1 — Supplementary Information [file 41467_2026_70705_MOESM1_ESM.pdf]

Supplementary Information

Table of Contents

Supplementary Figures

Supplementary Figure 1. Differential abundance of bacterial families along the gut

Supplementary Figure 2. Functional pathway differences along the gut.

Supplementary Figure 3. Species considered for each analysis

Supplementary Figure 4. Inoculum π and average mouse π

Supplementary Figure 5. Relative strain frequencies along the guts of humanized mice

Supplementary Figure 6. Variance in major strain frequency partitioned between gut region, mouse, and cage in humanized mice

Supplementary Figure 7. Variance in strain frequencies within and between cages

Supplementary Figure 8. Evolutionary changes along the guts of humanized mice

Supplementary Figure 9. Increase in taxonomic diversity and change in community membership along the length of the guts of conventional mice.

Supplementary Figure 10. Relative strain frequencies along the guts of conventional mice

Supplementary Figure 11. Variance in major strain frequency partitioned between gut region, mouse, and cage in conventional mice.

Supplementary Figure 12. Relative strain frequencies along the guts of healthy humans

Supplementary Figure 13. Evolutionary changes along the guts of healthy humans.

Supplementary Figure 14. Inferring strain frequency of *Bacteroides vulgatus* strains

Supplementary Figure 15. Supervised strain frequency inference of *Bacteroides uniformis* strains

Supplementary Figures

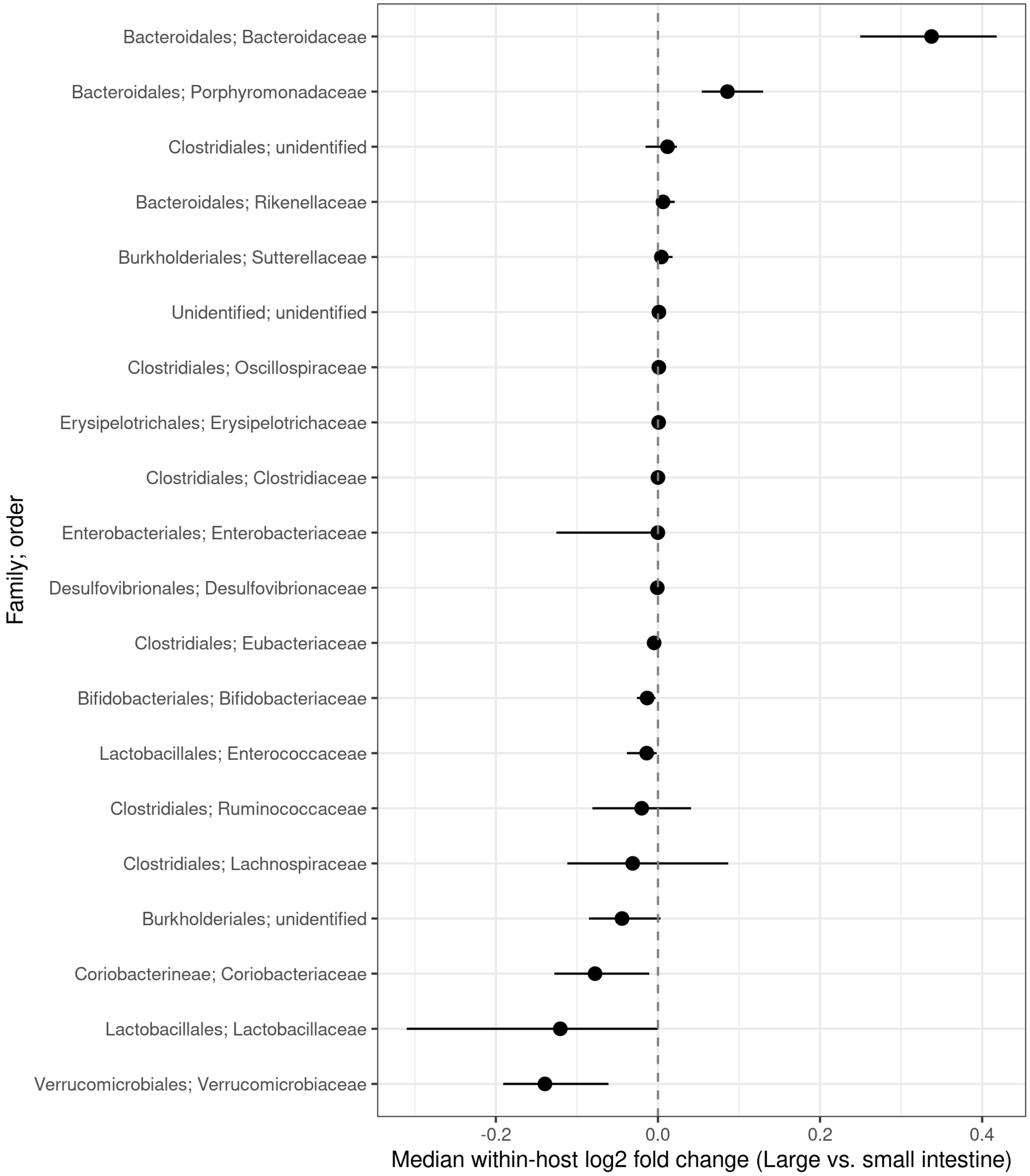

Supplementary Figure 1. Families have differential relative abundance along the gut. A paired Wilcoxon signed-rank test was used to estimate median log2 fold change (black dots) between paired family relative abundance values corresponding to the large and small intestine of the same mice (Methods). Error bars represent the 95% confidence intervals of the log2 fold change value estimates, as constructed by the Wilcoxon signed-rank test. Taxa labels represent

Order; *Family*, and are ordered from highest to lowest log2fold change. Positive log2 fold change values indicate families that are enriched in the large intestine, whereas negative log2 fold change values indicate families that are enriched in the small intestine.

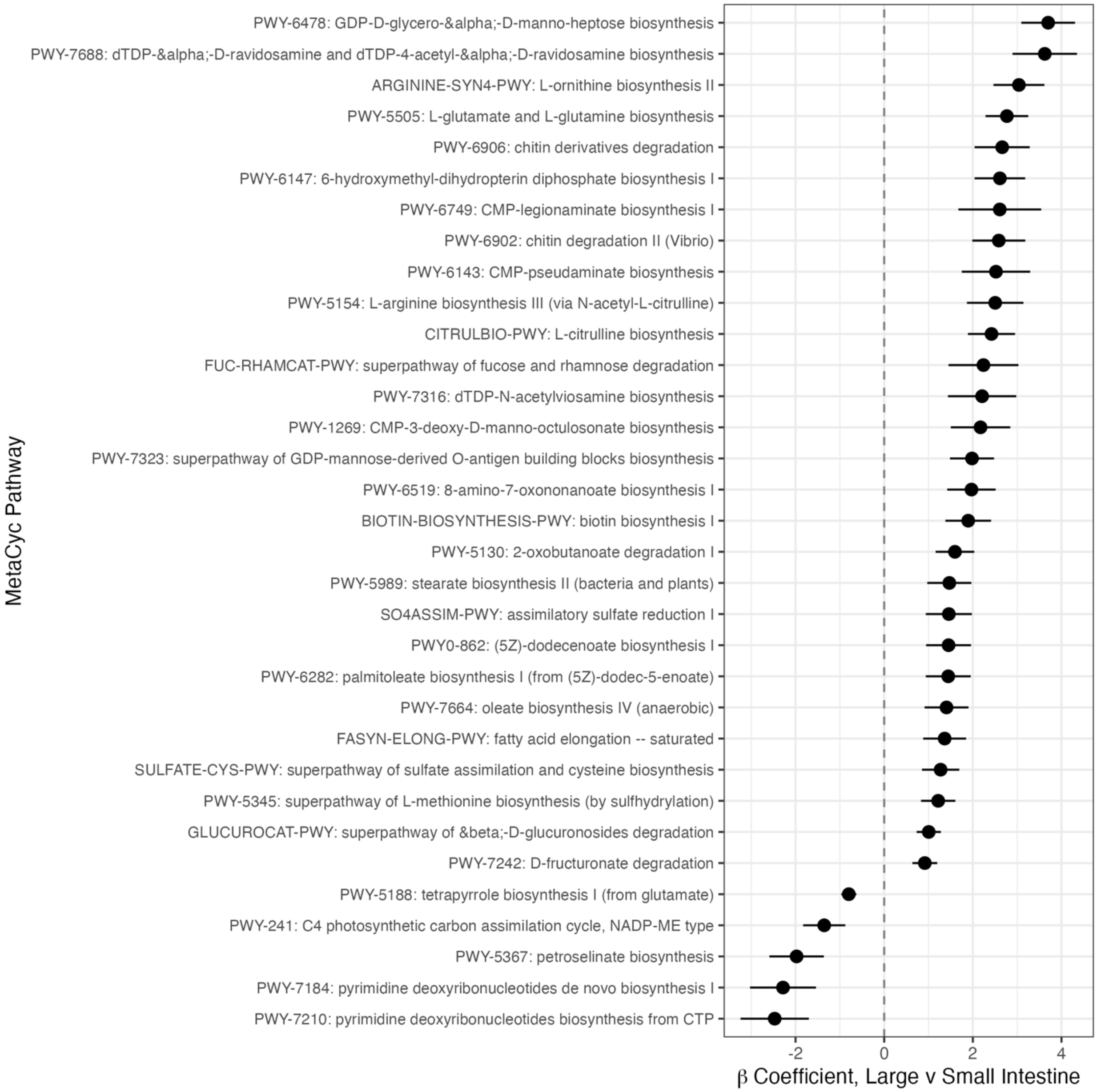

Supplementary Figure 2. Functional pathway differences along the gut. Estimated coefficients for the effect of gut location on $\log_2$ relative abundances from a MaAsLIN 3 multivariate linear model. Shown are 34 MetaCyc pathways with highly significant association ($q < 0.001$), none of which were associated with cage or read count ($q > 0.1$). Gut location was defined as a binary variable indicating either the large or small intestine. Positive coefficients indicate families that are enriched in the large intestine, whereas negative coefficients indicate

families that are enriched in the small intestine. Error bars represent 95% confidence intervals of the coefficient estimates.

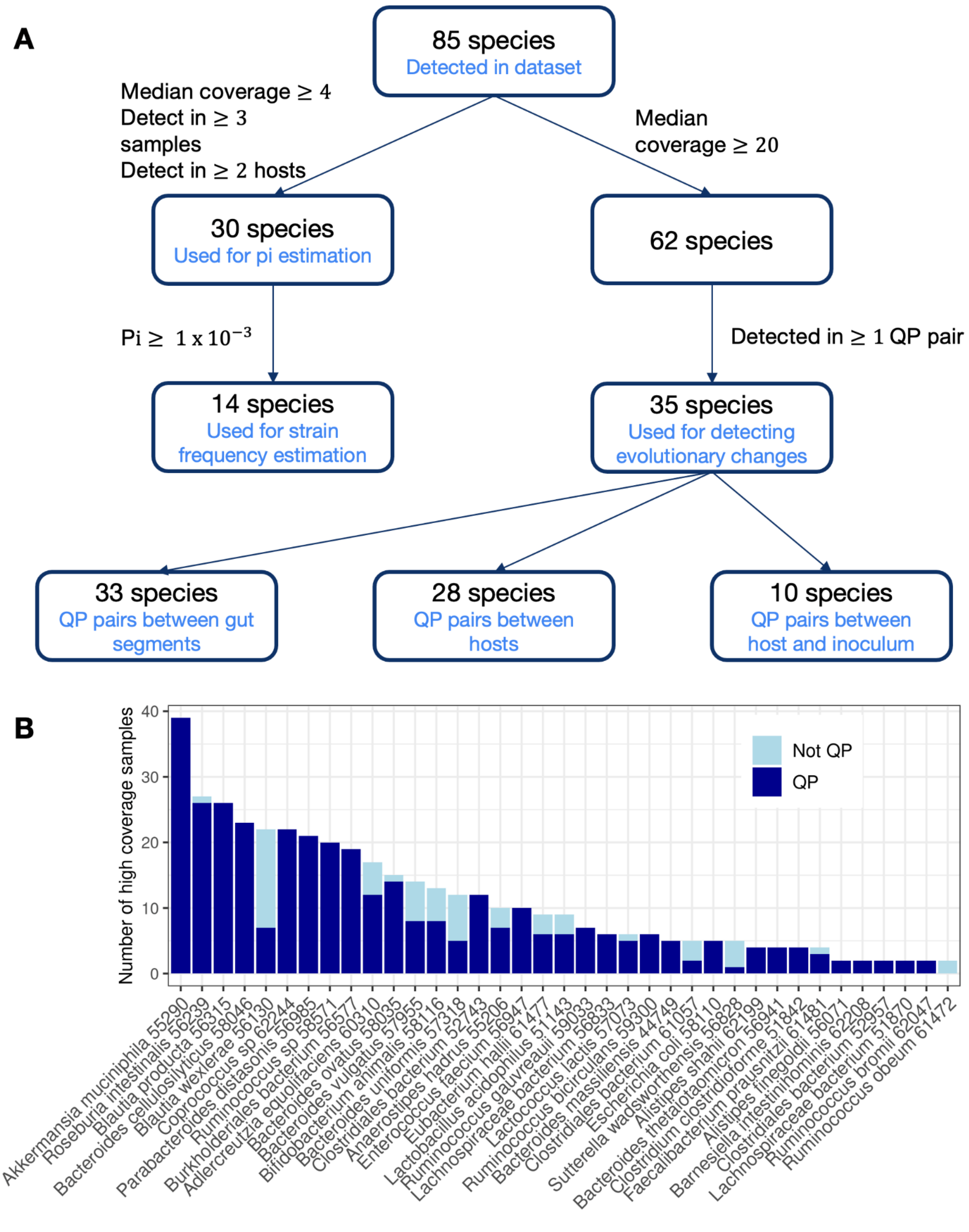

Supplementary Figure 3. Species considered for each analysis. (A) Species requirements for assessing within-species diversity with pi, inferring strain frequencies, and assessing evolutionary changes within hosts, between hosts and between inoculum and host. **(B)** Number of quasi-phaseable (QP) and non-quasi-phaseable (non-QP) genome pairs for all species in the dataset that have at least two high coverage samples (i.e., median coverage ≥ 20).

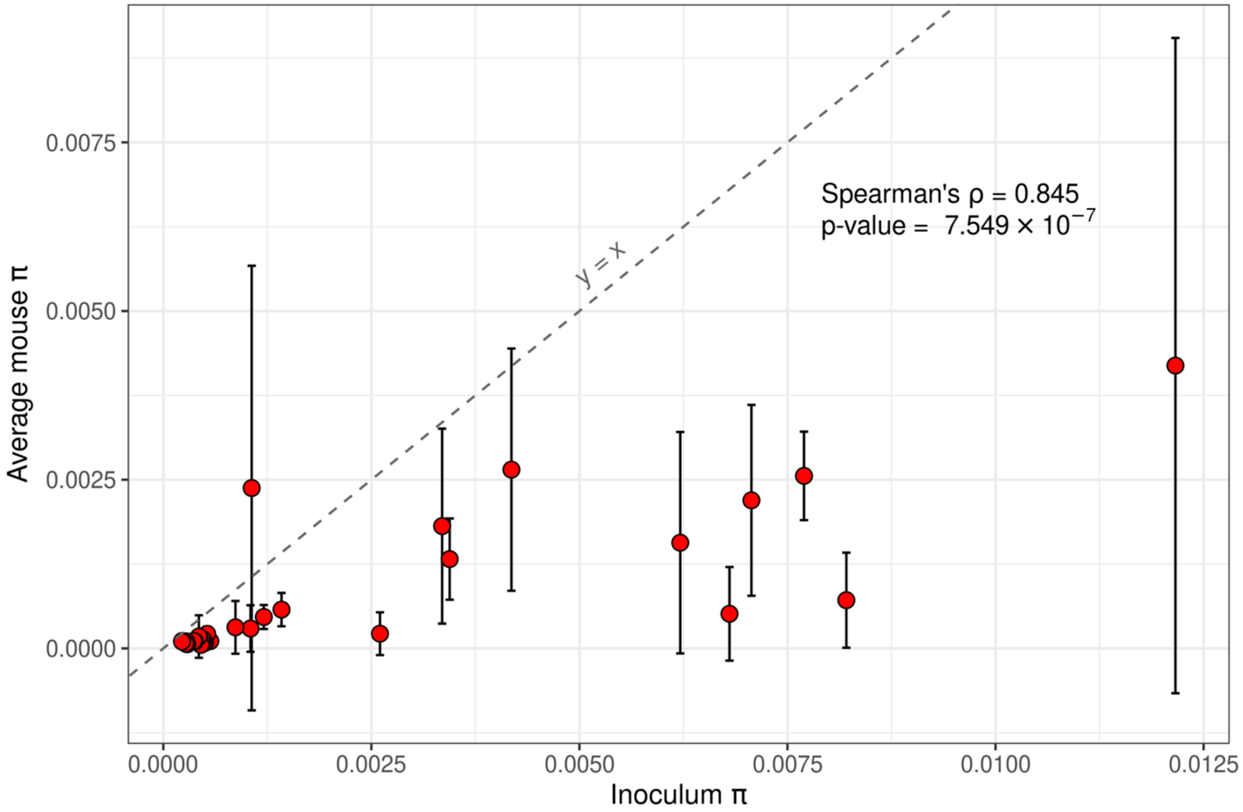

Supplementary Figure 4. Inoculum π and average mouse π. Nucleotide diversity (π) observed in the inoculum (x axis) and average nucleotide diversity observed across mouse samples (y axis) is visualized for the 30 most abundant and prevalent species in the humanized mouse cohort (Methods). Red dots represent the nucleotide diversity estimate, while error bars represent the standard deviation of π values observed in mouse samples. The dashed grey line represents the identity line (y = x).

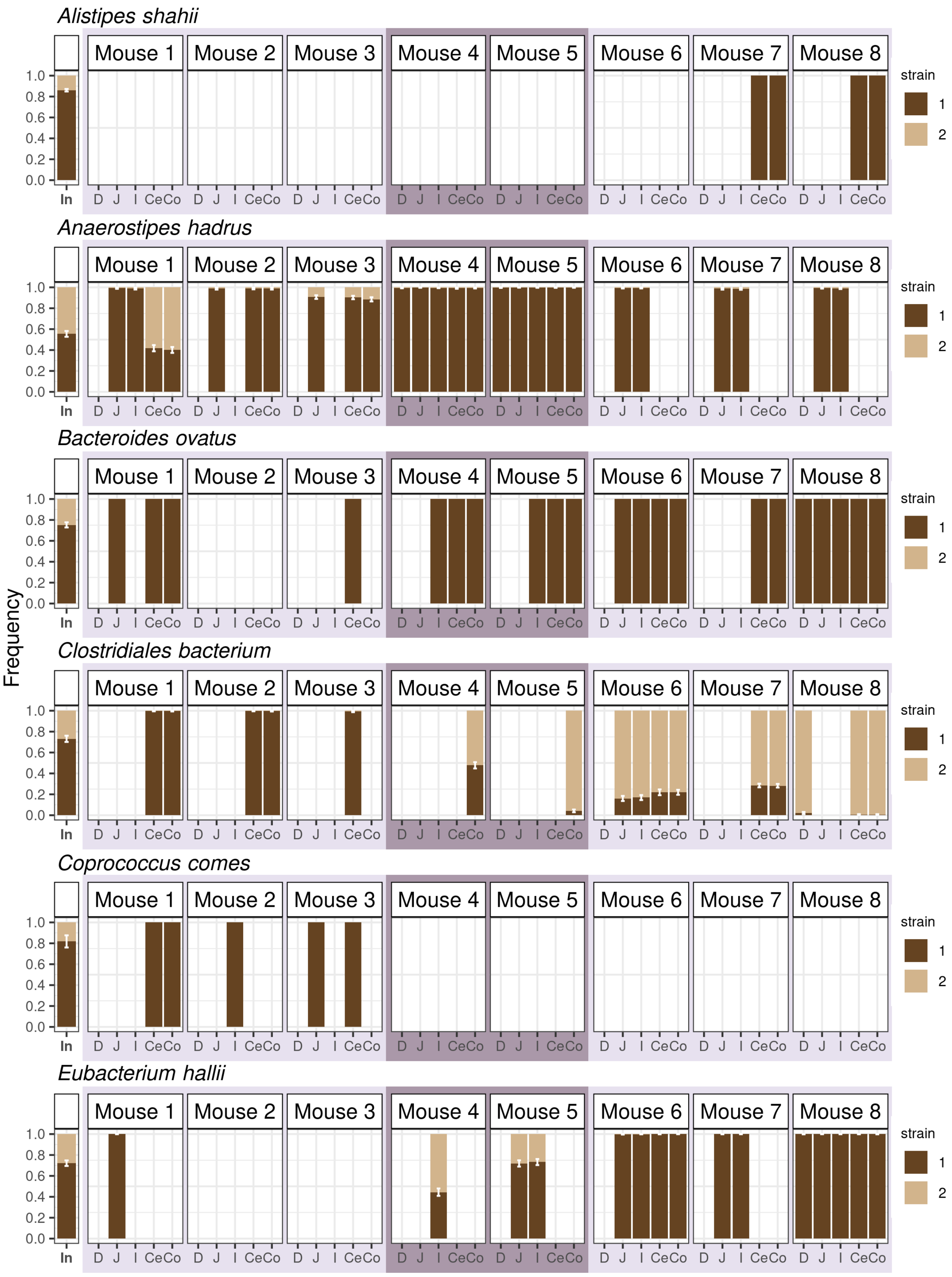

Alistipes shahii
Anaerostipes hadrus
Bacteroides ovatus
Clostridiales bacterium
Coprococcus comes
Eubacterium hallii
Mouse 1
Mouse 2
Mouse 3
Mouse 4
Mouse 5
Mouse 6
Mouse 7
Mouse 8
Frequency
1.0
0.8
0.6
0.4
0.2
0.0
In
D J I CeCo
strain
1
2

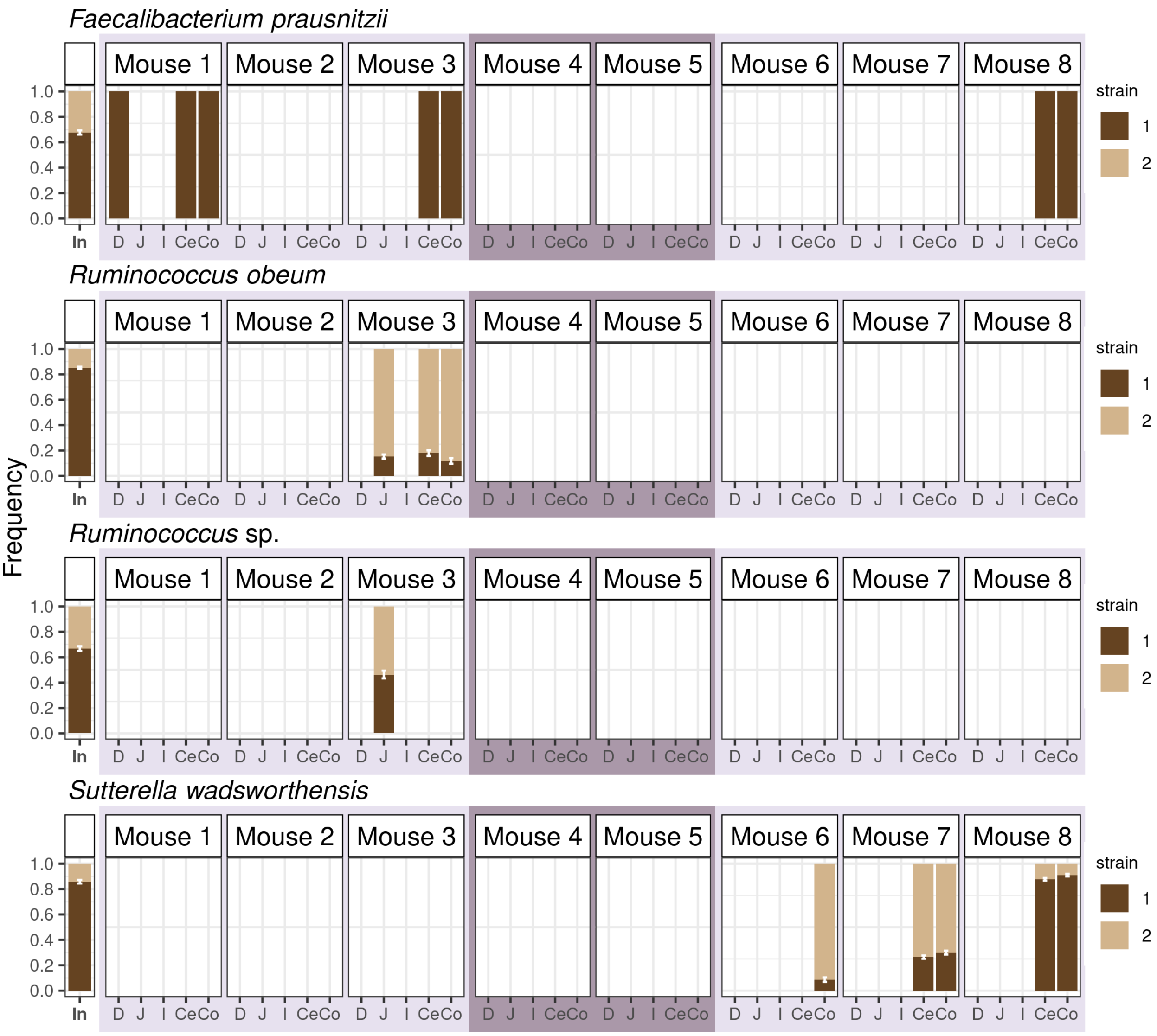

Supplementary Figure 5. Strain frequency along the guts of humanized mice. Strain frequency of co-colonizing strains was inferred across all samples for 10 species that had $\pi \geq 1 \times 10^{-3}$ in the inoculum. Strain 1 and 2 frequencies are shown in dark and light brown, respectively. Strain frequency is indicated on the y-axis, with error bars representing the 95% confidence intervals for the inferred strain frequency (Methods). Cages 1-3 are delineated with alternating light and dark purple boxes. Strain frequencies of the four species not shown here can be found in Figure 4 of the main text.

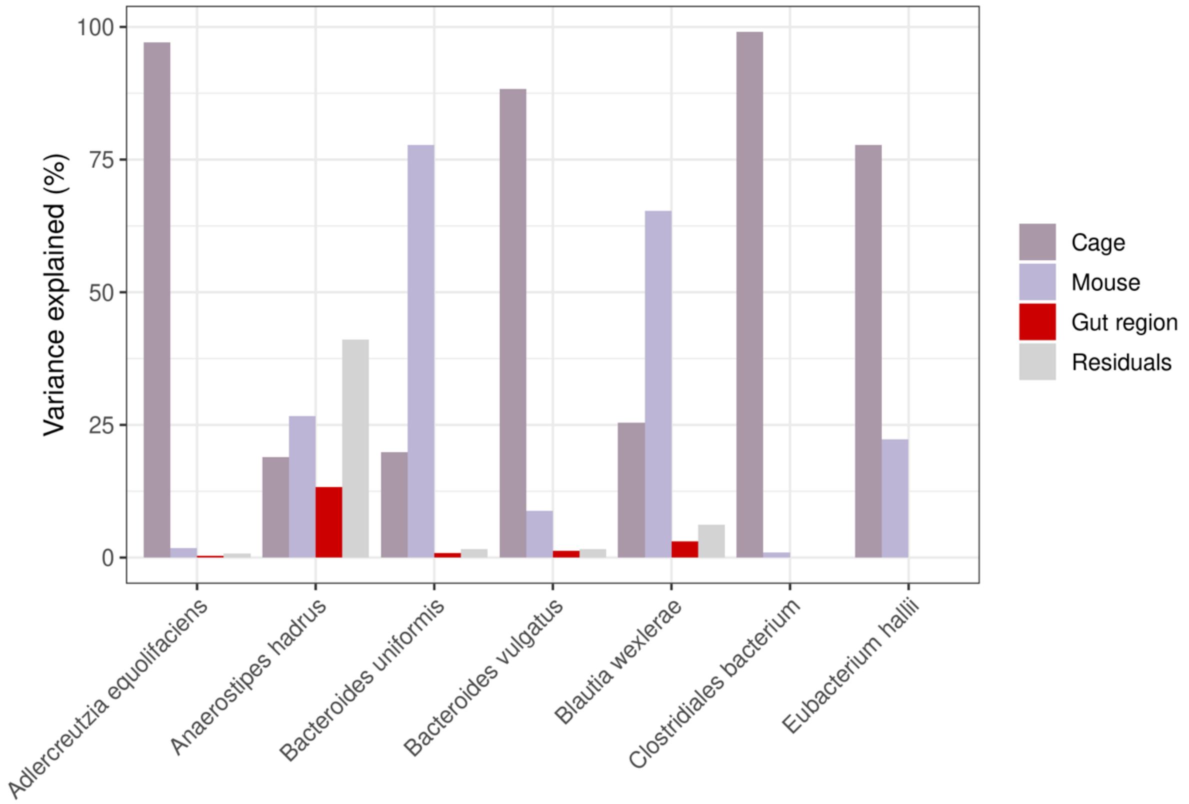

Supplementary Figure 6. Variance in major strain frequency partitioned between gut region, mouse, and cage in humanized mice. ANOVA was used to quantify the amount of variance in major strain relative frequency explained by “cage”, “mouse”, and “gut region” in the seven species for which enough high coverage samples were available to test the effect of all three variables (Methods). Residuals of the ANOVA represent unexplained variance.

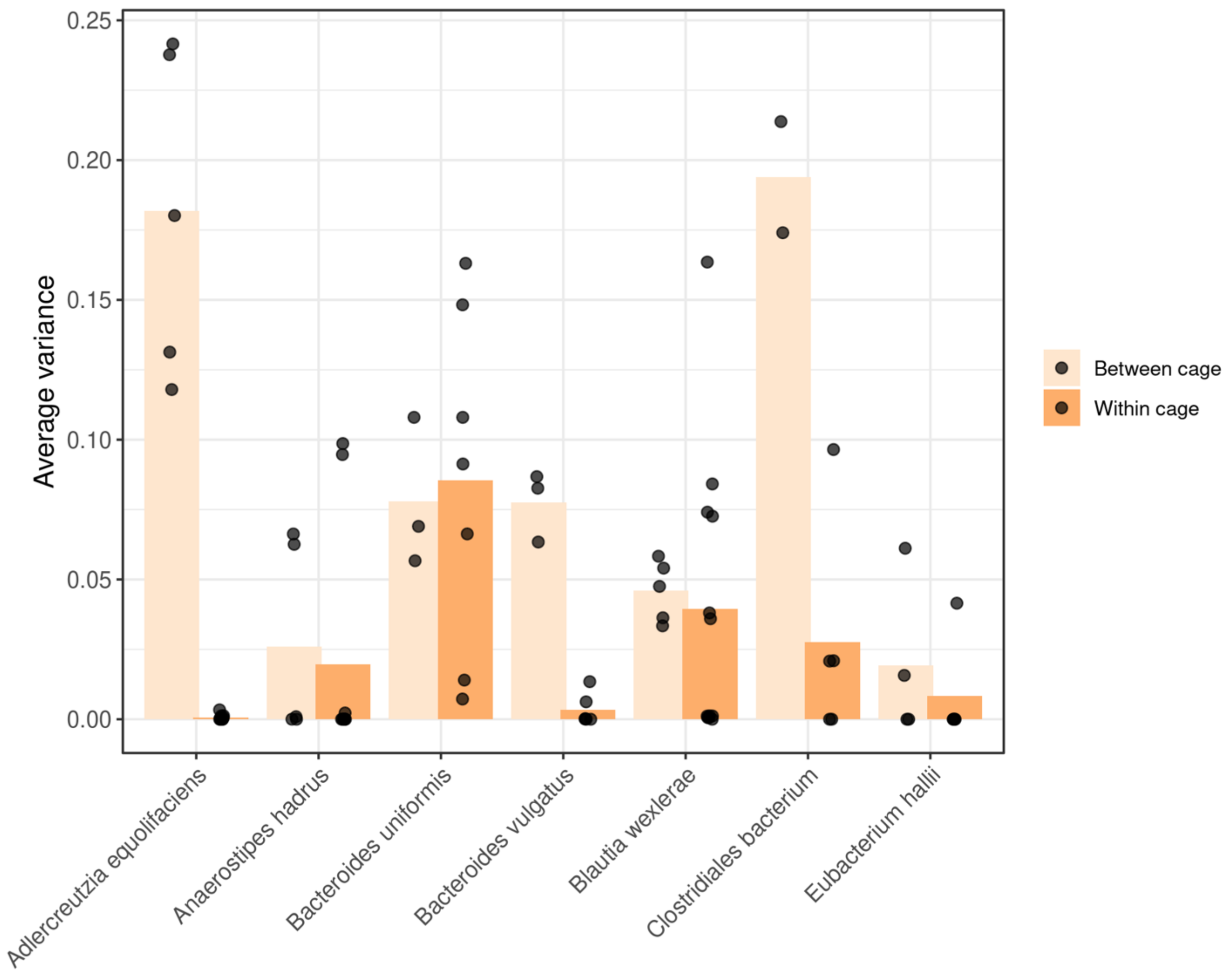

Supplementary Figure 7. Variance in strain frequencies within and between cages. Variance in major strain frequency was measured for samples belonging to the same gut region in different mice within the same cage ("Within cage," dark orange) and between different cages ("Between cage," light orange). Within cage bars represent the average within cage variance across the three cages and five gut regions, while between cage bars represent between cage variance averaged across the five gut regions. Each black dot represents the variance in strain frequency across the same gut region (e.g., cecum) in different mice within or between cages, respectively.

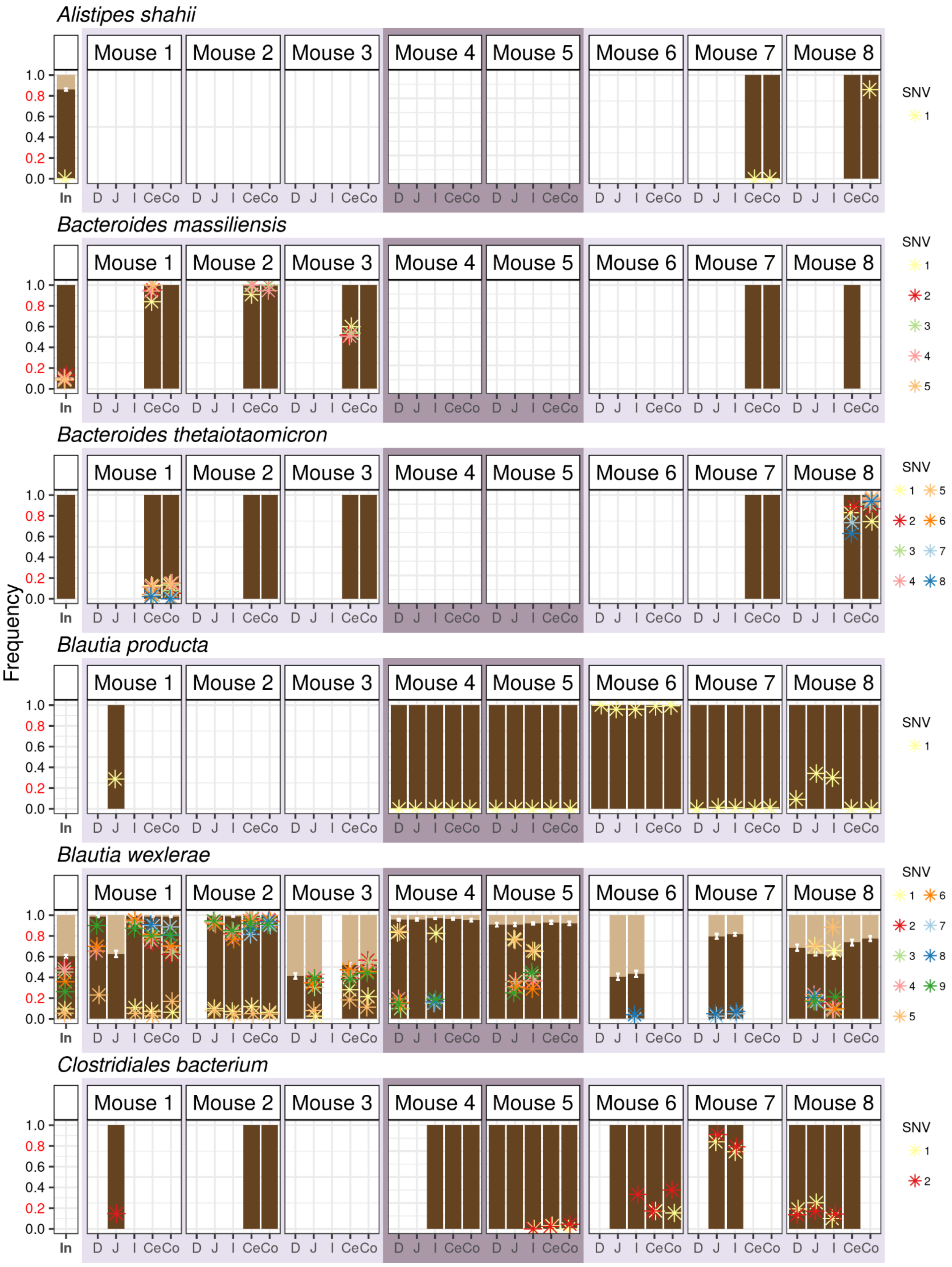
Alistipes shahii
Bacteroides massiliensis
Bacteroides thetaiotaomicron
Blautia producta
Blautia wexlerae
Clostridiales bacterium
Mouse 1
Mouse 2
Mouse 3
Mouse 4
Mouse 5
Mouse 6
Mouse 7
Mouse 8
Frequency
1.0
0.8
0.6
0.4
0.2
0.0
In
D J I CeCo
SNV
1
2
3
4
5
6
7
8
9

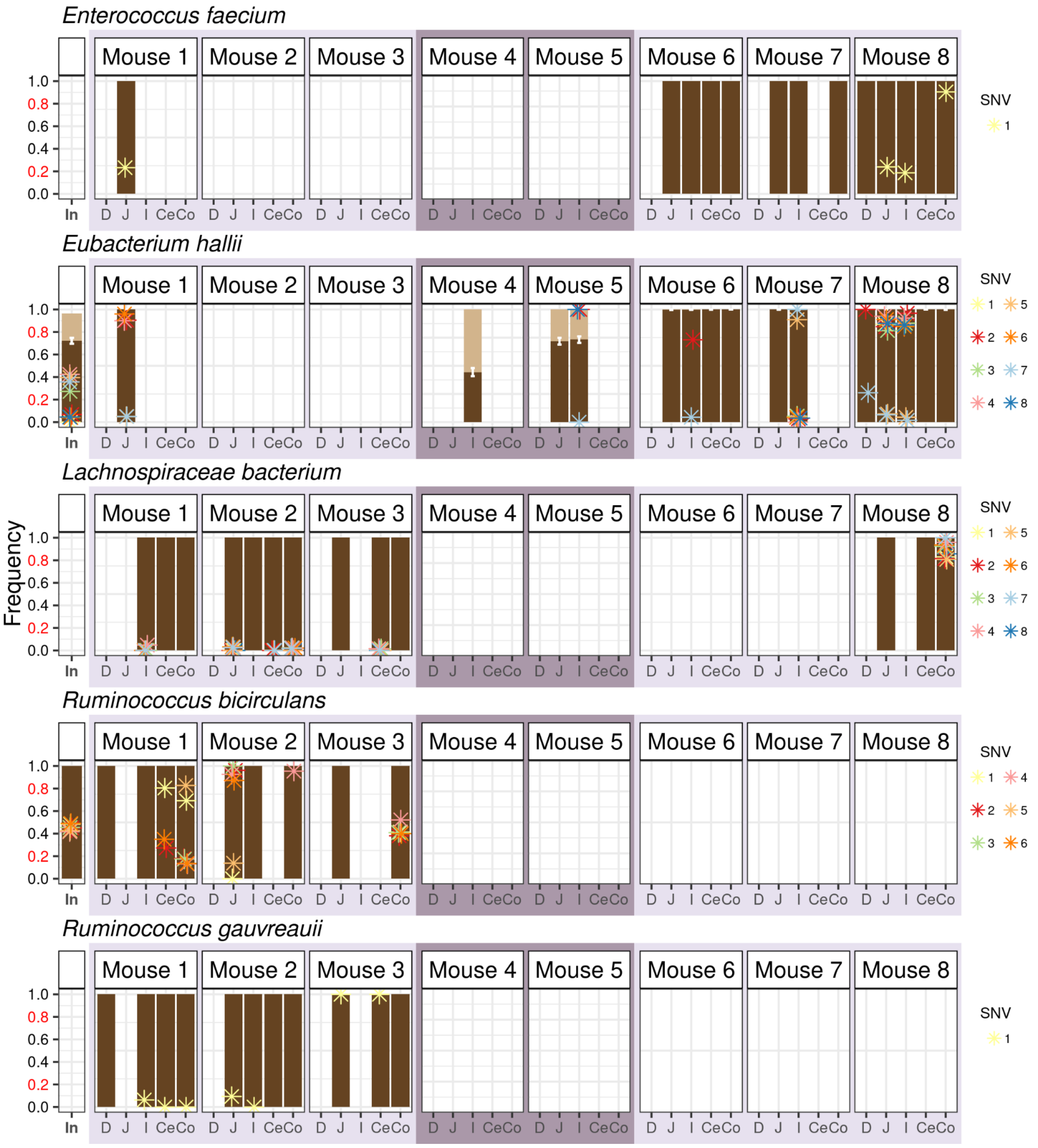

Supplementary Figure 8. Evolutionary changes along the guts of humanized mice. SNVs in 13 species underwent extreme allele frequency changes (i.e., $f \leq 0.2$ to $f \geq 0.8$) between pairs of QP samples. Allele frequencies for these SNVs were calculated in all samples for which the loci of interest had a coverage $D \geq 20$. Asterisks represent the allele frequency of a given SNV, with each SNV within a species having its own unique color. Samples lack asterisks for particular SNVs when those loci do not have adequate coverage to infer allele frequency. When multiple strains of the same species are present, frequencies of co-colonizing strains are

represented as dark and light shades of brown, and error bars represent the 95% confidence intervals for the inferred strain frequency

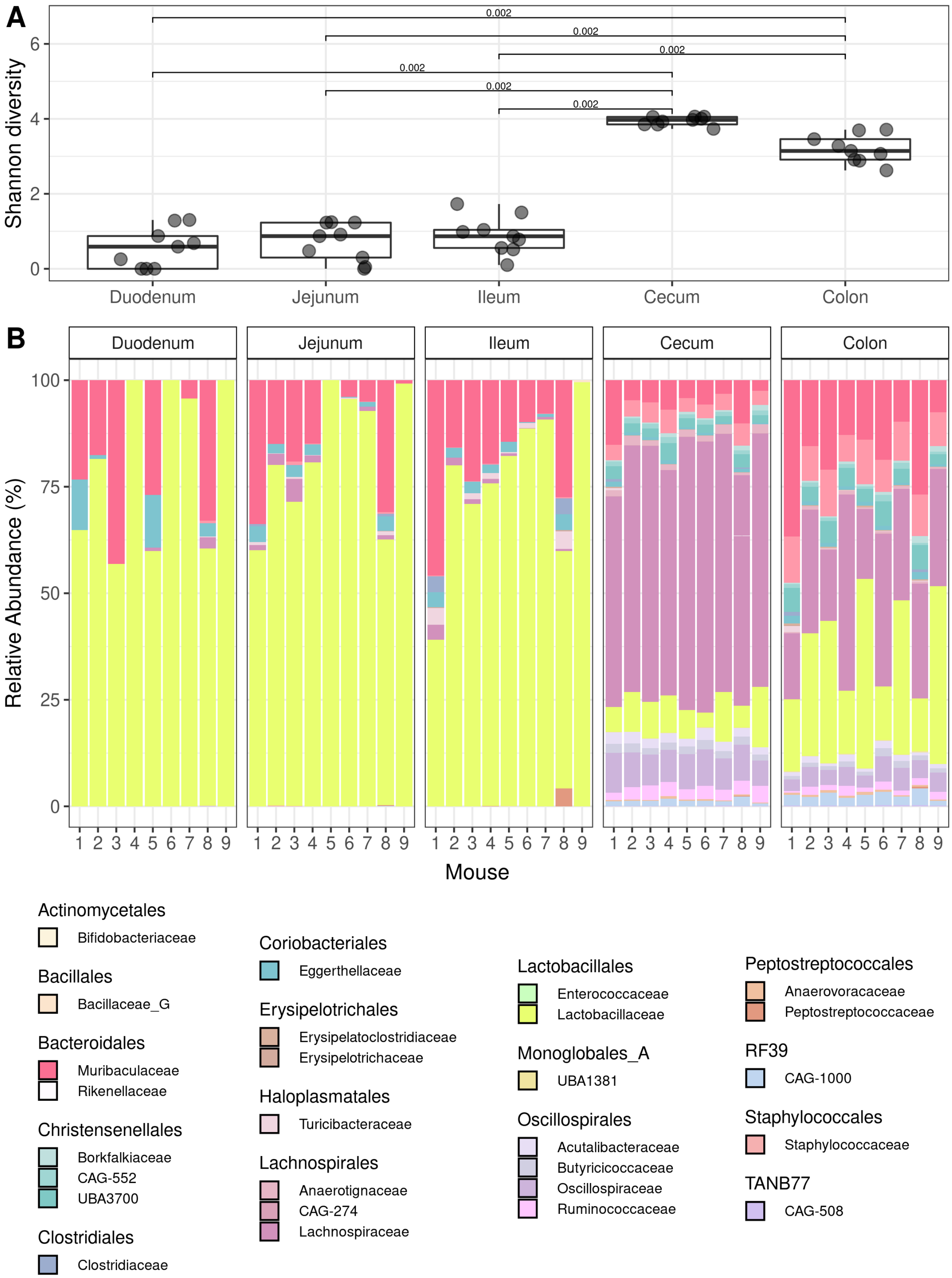

A
Shannon diversity
0.002
0.002
0.002
0.002
0.002
0.002
0
2
4
6
Duodenum
Jejunum
Ileum
Cecum
Colon
B
Duodenum
Jejunum
Ileum
Cecum
Colon
Relative Abundance (%)
100
75
50
25
0
1 2 3 4 5 6 7 8 9
Mouse
Actinomycetales
Bifidobacteriaceae
Bacillales
Bacillaceae_G
Bacteroidales
Muribaculaceae
Rikenellaceae
Christensenellales
Borkfalkiaceae
CAG-552
UBA3700
Clostridiales
Clostridiaceae
Coriobacteriales
Eggerthellaceae
Erysipelotrichales
Erysipelatoclostridiaceae
Erysipelotrichaceae
Haloplasmatales
Turicibacteraceae
Lachnospirales
Anaerotignaceae
CAG-274
Lachnospiraceae
Lactobacillales
Enterococcaceae
Lactobacillaceae
Monoglobales_A
UBA1381
Oscillospirales
Acutalibacteraceae
Butyricicoccaceae
Oscillospiraceae
Ruminococcaceae
Peptostreptococcales
Anaerovoracaceae
Peptostreptococcaceae
RF39
CAG-1000
Staphylococcales
Staphylococcaceae
TANB77
CAG-508

Supplementary Figure 9. Increase in taxonomic diversity and change in community membership along the length of the guts of conventional mice. (A) Alpha diversity (Shannon Index) estimates for bacteria in different regions of the gut for all mice (n = 9). Two-sided paired Wilcoxon signed-rank tests were performed between all possible pairs of small intestinal versus large intestinal samples (n = 9 for each paired test, corresponding to 9 mice). P-values for each test are indicated over the brackets. **(B)** Relative abundance of bacterial families in the five gut regions and inoculum. Bacterial families are labeled with unique colors and grouped by order in the legend.

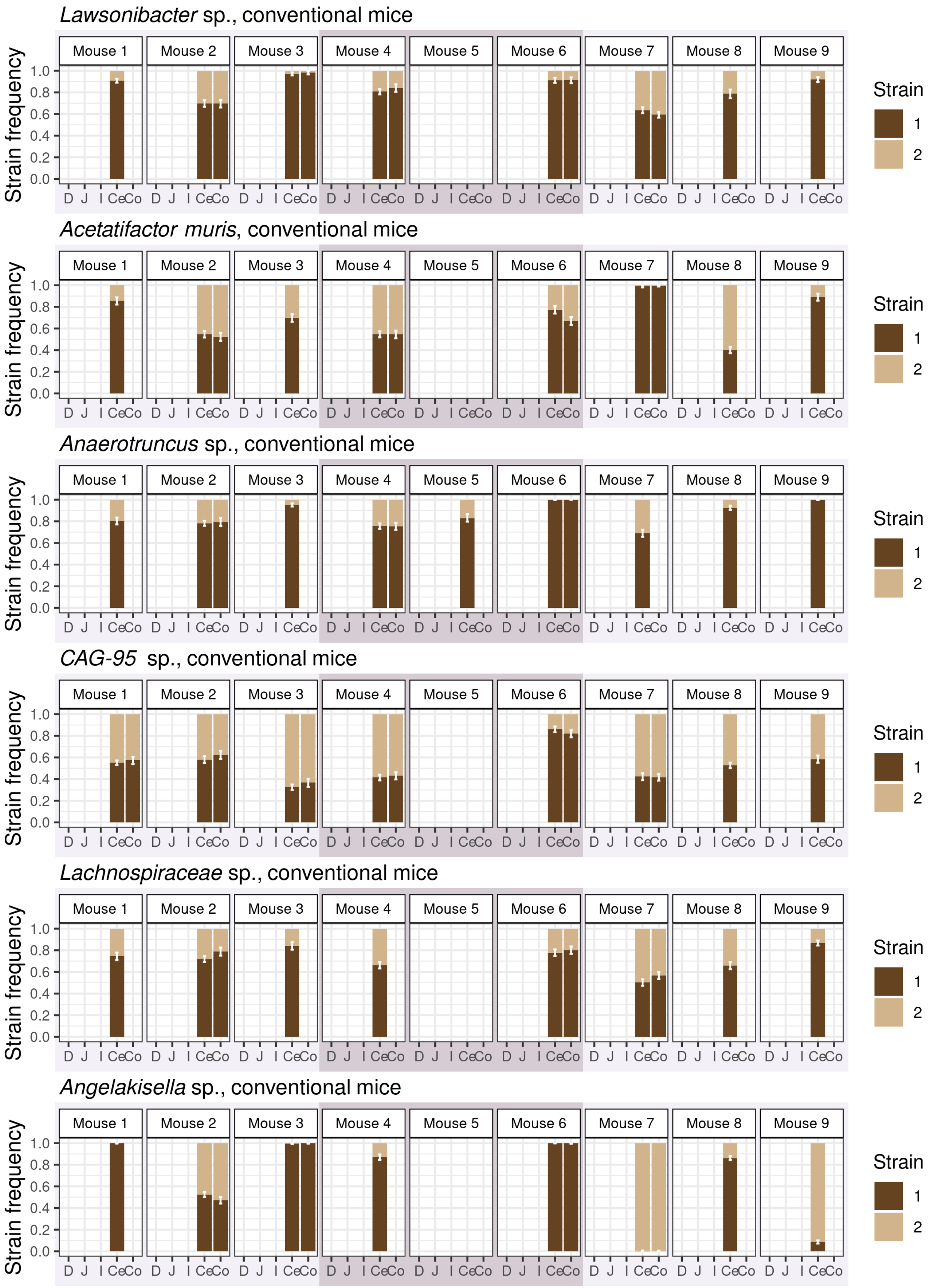

Lawsonibacter sp., conventional mice
Acetatifactor muris, conventional mice
Anaerotruncus sp., conventional mice
CAG-95 sp., conventional mice
Lachnospiraceae sp., conventional mice
Angelakisella sp., conventional mice
Mouse 1
Mouse 2
Mouse 3
Mouse 4
Mouse 5
Mouse 6
Mouse 7
Mouse 8
Mouse 9
Strain frequency
0.0
0.2
0.4
0.6
0.8
1.0
D J I Ce Co
Strain
1
2

Supplementary Figure 10. Strain frequency along the guts of conventional mice. Strain frequency of co-colonizing strains was inferred across all conventional mouse samples for six species shown here. Strain frequency is indicated on the y-axis, with strain 1 and 2 frequencies indicated by the dark and light brown bars, respectively, and error bars representing the bootstrapped 95% confidence intervals for the inferred strain frequency (Methods). Cages 1-3 are delineated with alternating light and dark purple boxes. Strain frequencies of the one species with multiple colonizing strains not shown here can be found in Figure 6A of the main text.

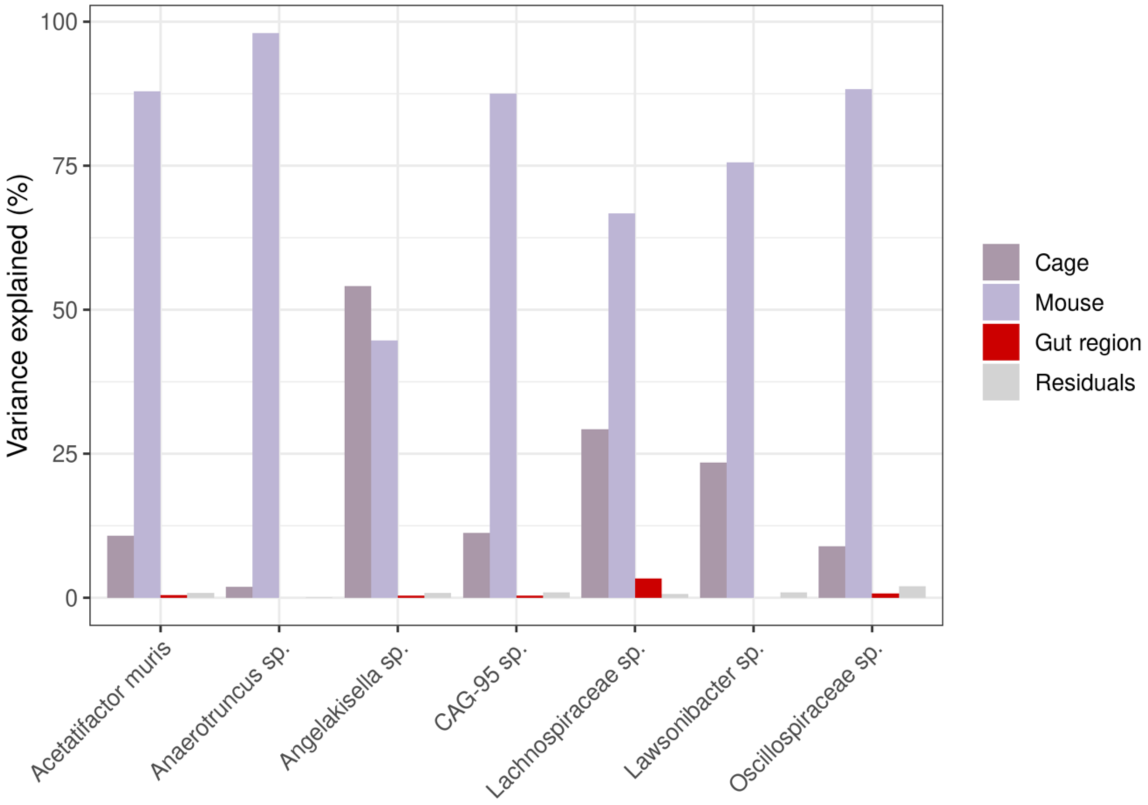

Supplementary Figure 11. Variance in major strain frequency partitioned between gut region, mouse, and cage in conventional mice. ANOVA was used to quantify the amount of variance in major strain relative frequency explained by “cage”, “mouse”, and “gut region” in the seven species for which enough high coverage samples were available to test the effect of all three variables (Methods). Residuals of the ANOVA represent unexplained variance.

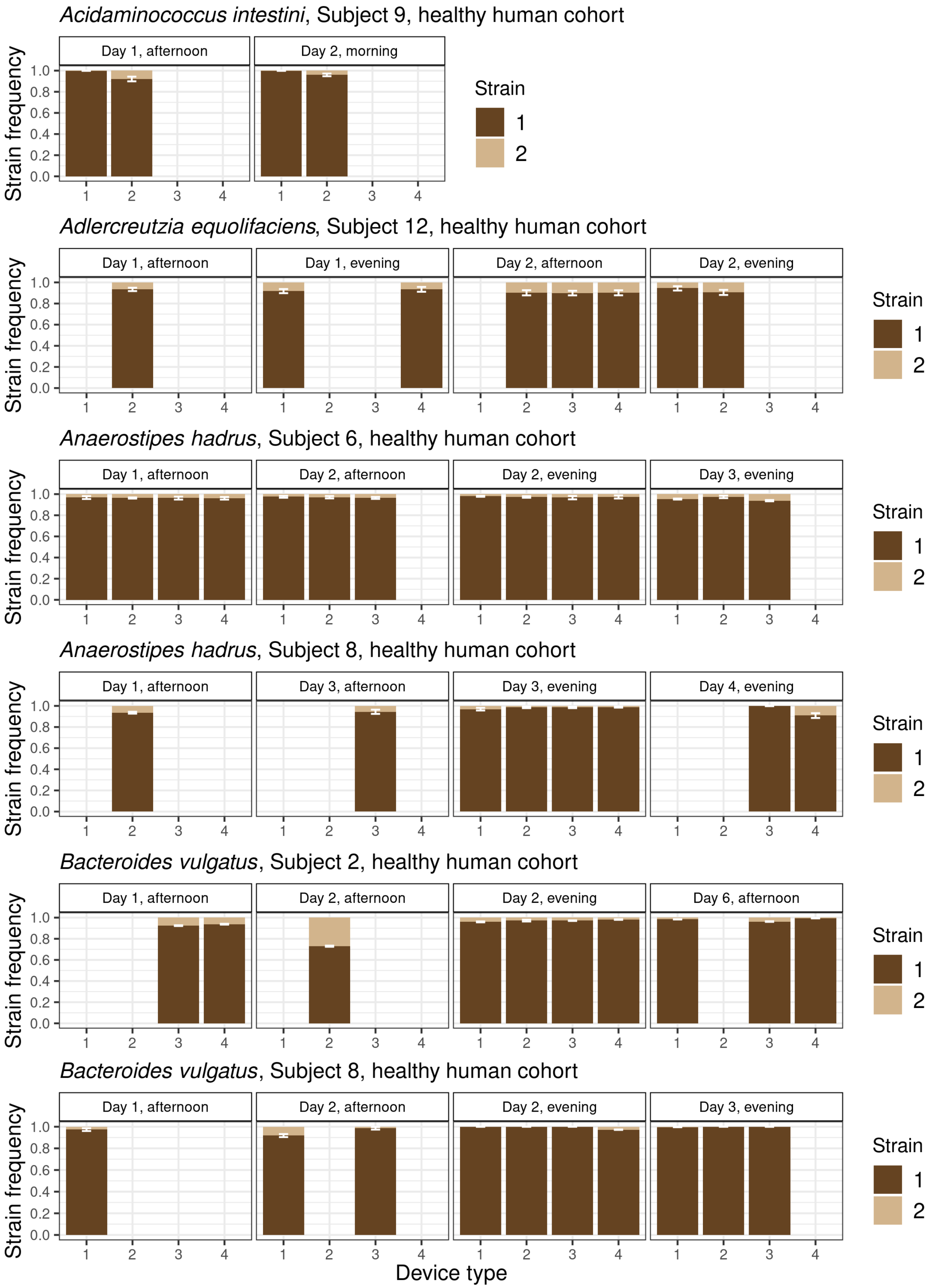

Acidaminococcus intestini, Subject 9, healthy human cohort
Day 1, afternoon
Day 2, morning
Adlercreutzia equolifaciens, Subject 12, healthy human cohort
Day 1, afternoon
Day 1, evening
Day 2, afternoon
Day 2, evening
Anaerostipes hadrus, Subject 6, healthy human cohort
Day 1, afternoon
Day 2, afternoon
Day 2, evening
Day 3, evening
Anaerostipes hadrus, Subject 8, healthy human cohort
Day 1, afternoon
Day 3, afternoon
Day 3, evening
Day 4, evening
Bacteroides vulgatus, Subject 2, healthy human cohort
Day 1, afternoon
Day 2, afternoon
Day 2, evening
Day 6, afternoon
Bacteroides vulgatus, Subject 8, healthy human cohort
Day 1, afternoon
Day 2, afternoon
Day 2, evening
Day 3, evening
Strain frequency
0.0
0.2
0.4
0.6
0.8
1.0
1
2
3
4
Device type
Strain
1
2

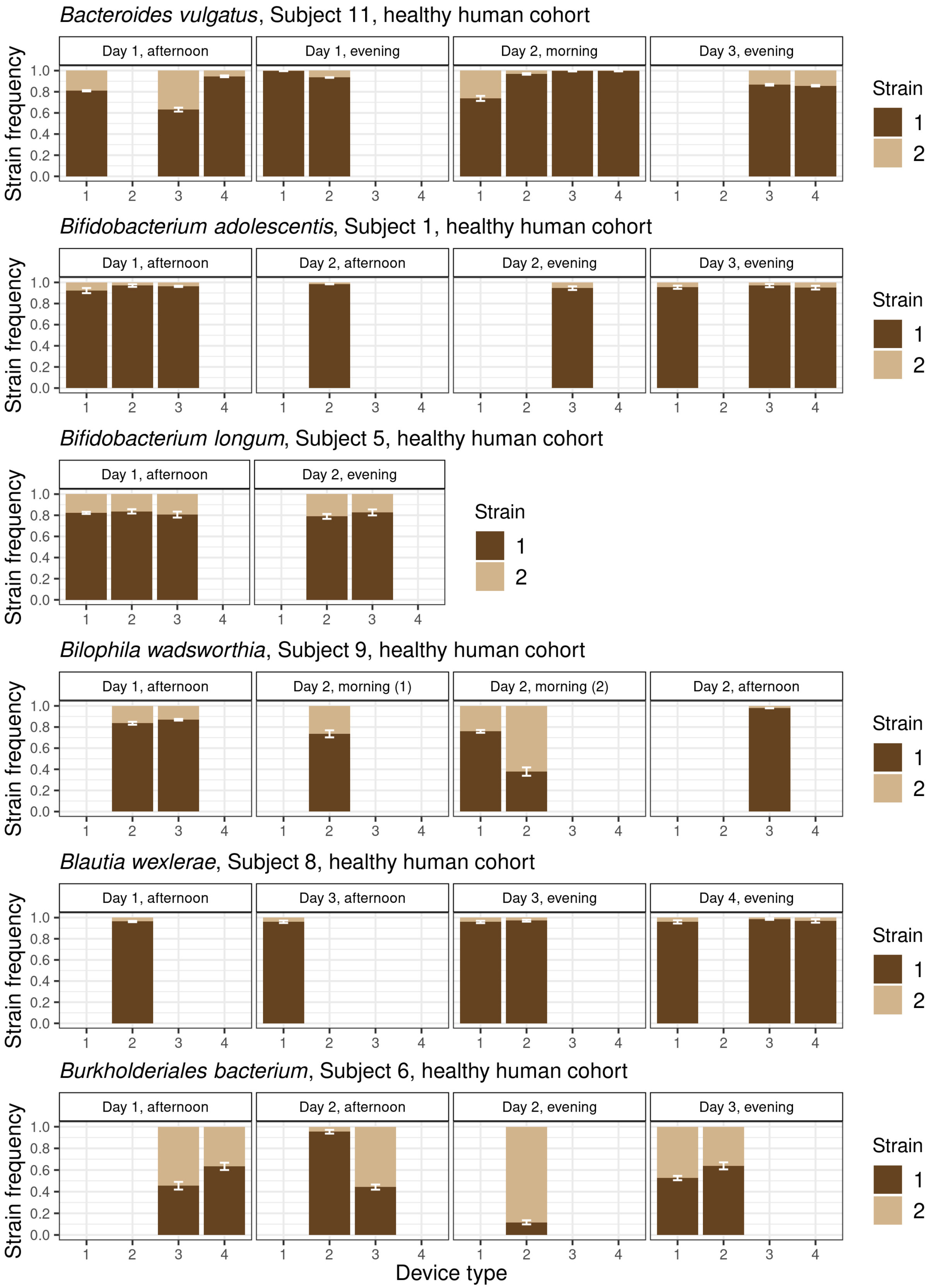

Bacteroides vulgatus, Subject 11, healthy human cohort
Day 1, afternoon
Day 1, evening
Day 2, morning
Day 3, evening
Bifidobacterium adolescentis, Subject 1, healthy human cohort
Day 1, afternoon
Day 2, afternoon
Day 2, evening
Day 3, evening
Bifidobacterium longum, Subject 5, healthy human cohort
Day 1, afternoon
Day 2, evening
Bilophila wadsworthia, Subject 9, healthy human cohort
Day 1, afternoon
Day 2, morning (1)
Day 2, morning (2)
Day 2, afternoon
Blautia wexlerae, Subject 8, healthy human cohort
Day 1, afternoon
Day 3, afternoon
Day 3, evening
Day 4, evening
Burkholderiales bacterium, Subject 6, healthy human cohort
Day 1, afternoon
Day 2, afternoon
Day 2, evening
Day 3, evening
Strain frequency
0.0
0.2
0.4
0.6
0.8
1.0
1
2
3
4
Device type
Strain
1
2

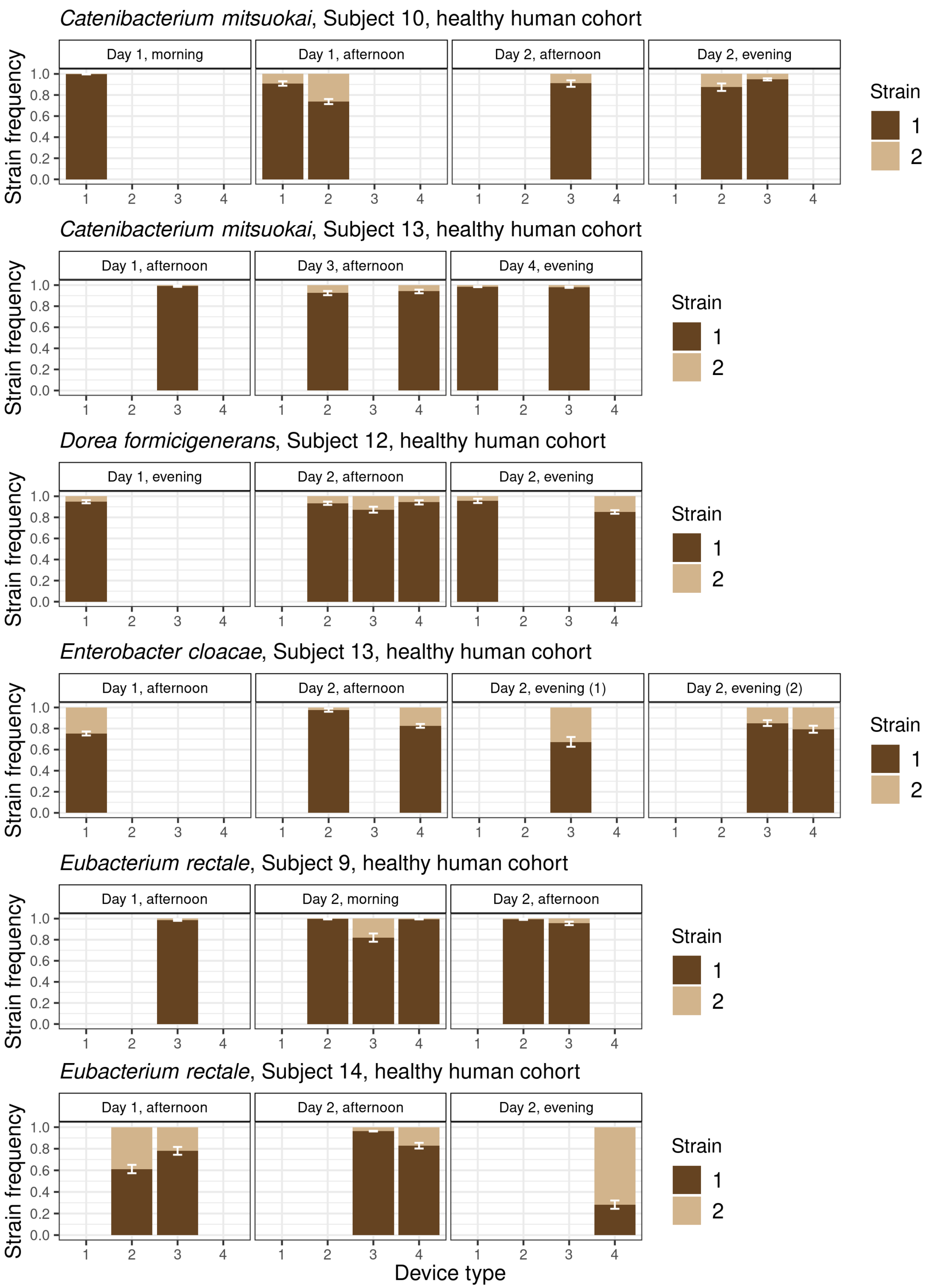
Catenibacterium mitsuokai, Subject 10, healthy human cohort
Day 1, morning
Day 1, afternoon
Day 2, afternoon
Day 2, evening
Catenibacterium mitsuokai, Subject 13, healthy human cohort
Day 1, afternoon
Day 3, afternoon
Day 4, evening
Dorea formicigenerans, Subject 12, healthy human cohort
Day 1, evening
Day 2, afternoon
Day 2, evening
Enterobacter cloacae, Subject 13, healthy human cohort
Day 1, afternoon
Day 2, afternoon
Day 2, evening (1)
Day 2, evening (2)
Eubacterium rectale, Subject 9, healthy human cohort
Day 1, afternoon
Day 2, morning
Day 2, afternoon
Eubacterium rectale, Subject 14, healthy human cohort
Day 1, afternoon
Day 2, afternoon
Day 2, evening
Strain frequency
0.0
0.2
0.4
0.6
0.8
1.0
1
2
3
4
Device type
Strain
1
2

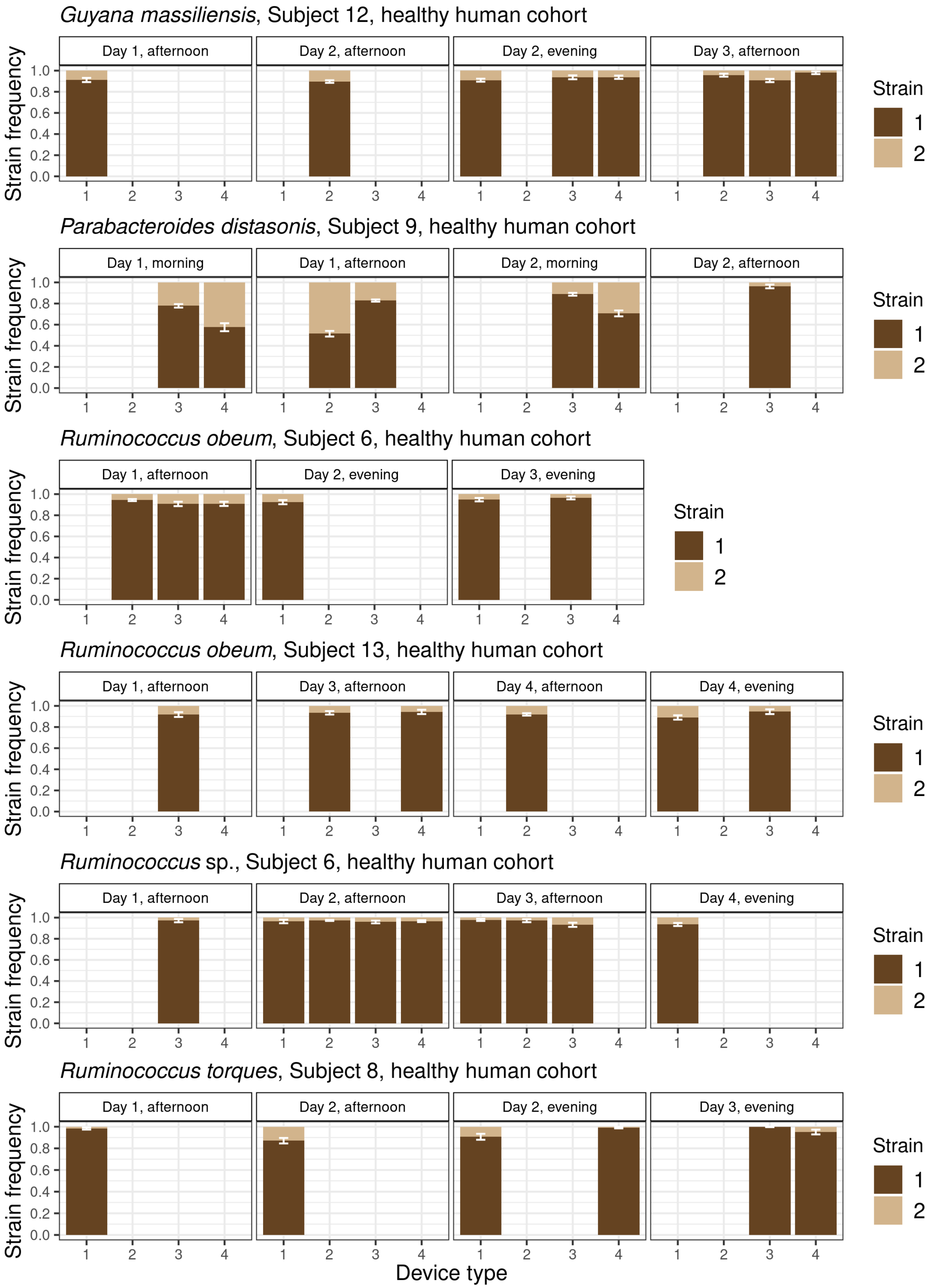

Guyana massiliensis, Subject 12, healthy human cohort
Day 1, afternoon
Day 2, afternoon
Day 2, evening
Day 3, afternoon
Parabacteroides distasonis, Subject 9, healthy human cohort
Day 1, morning
Day 1, afternoon
Day 2, morning
Day 2, afternoon
Ruminococcus obeum, Subject 6, healthy human cohort
Day 1, afternoon
Day 2, evening
Day 3, evening
Ruminococcus obeum, Subject 13, healthy human cohort
Day 1, afternoon
Day 3, afternoon
Day 4, afternoon
Day 4, evening
Ruminococcus sp., Subject 6, healthy human cohort
Day 1, afternoon
Day 2, afternoon
Day 3, afternoon
Day 4, evening
Ruminococcus torques, Subject 8, healthy human cohort
Day 1, afternoon
Day 2, afternoon
Day 2, evening
Day 3, evening
Strain frequency
0.0
0.2
0.4
0.6
0.8
1.0
1
2
3
4
Device type
Strain
1
2

Supplementary Figure 12. Strain frequency along the guts of healthy humans. Strain frequency of species with co-colonizing strains was inferred in a cohort of healthy human subjects. These subject swallowed capsule devices that collected luminal contents along their guts, where device type 1 targeted the pyloric sphincter to the upper small intestine, device type 2 targeted the upper to mid-small intestine, device type 3 targeted the mid- to lower small intestine, and device type 4 targeted the lower small intestine into the ascending colon. Visualized here are the 24 species x host pairs (representing 18 species across 11 subjects) which met prevalence filters (Methods). If strain frequency was inferred in more than four timepoints, four were manually chosen for visualization here. Strain frequency is indicated on the y-axis with strain 1 and 2 frequencies indicated by the dark and light brown bars, respectively, and with error bars representing the bootstrapped 95% confidence intervals for the inferred strain frequency (Methods). Timepoints represent the time at which capsules were swallowed, with days being relative to the first timepoint plotted and times of day being coarsened into "morning" (before 12 pm PST), "afternoon" (after 12pm and before 8 pm PST), and "evening" (after 8 pm PST) bins (all dates and times have been converted to PST from UTC, which is how they were formatted in the original metadata from Shalon et al.). Strain frequencies of the one species x host pair with multiple colonizing strains not shown here can be found in Figure 6D of the main text.

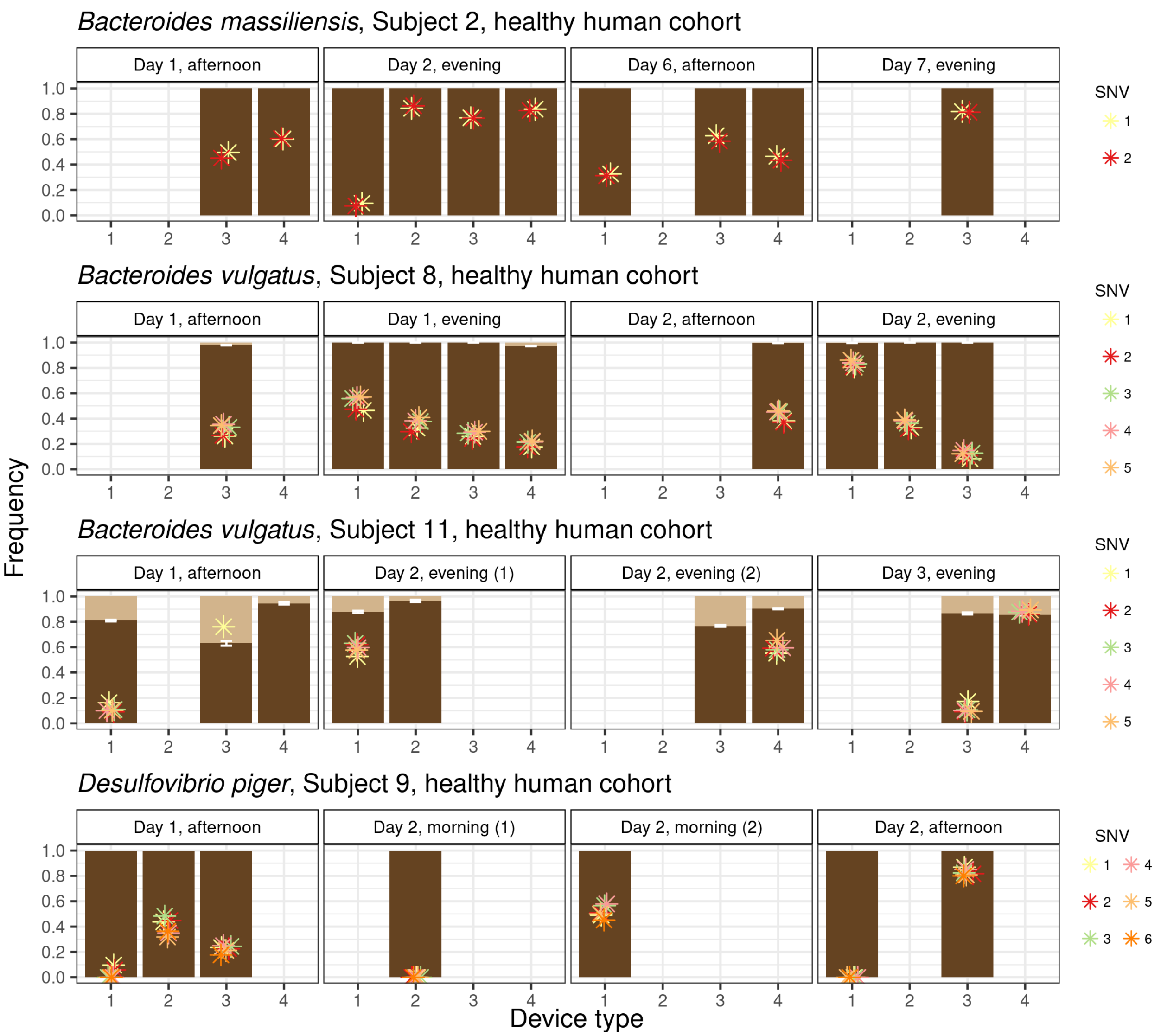

Supplementary Figure 13. Evolutionary changes along the guts of healthy humans. We visualized SNVs undergoing extreme allele frequency changes (i.e., $f \leq 0.2$ to $f \geq 0.8$ or vice versa) in at least timepoint in species x host that had sufficient temporal and spatial data (At least two timepoints with at least two device types per timepoint). These filters yielded 18 SNVs detected in 4 species x host pairs, representing 3 unique species across 4 unique hosts. Allele frequencies for these SNVs were calculated in all samples for which the loci of interest had a coverage $D \geq 20$. Asterisks represent the allele frequency of a given SNV, with each SNV within a species having its own unique color. Samples lack asterisks for SNVs when those SNVs do not have adequate coverage to infer allele frequency. When multiple strains of the same species are present, frequencies of co-colonizing strains are represented as dark and light shades of brown, and error bars represent the bootstrapped 95% confidence intervals of the inferred strain frequencies. Timepoints represent the time at which capsules were swallowed, with days being relative to the first timepoint plotted and times of day being coarsened into "morning" (before 12 pm PST), "afternoon" (after 12pm and before 8 pm PST), and "evening" (after 8 pm

PST) bins (all dates and times have been converted to PST from UTC, which is how they were formatted in the original metadata from Shalon et al.).

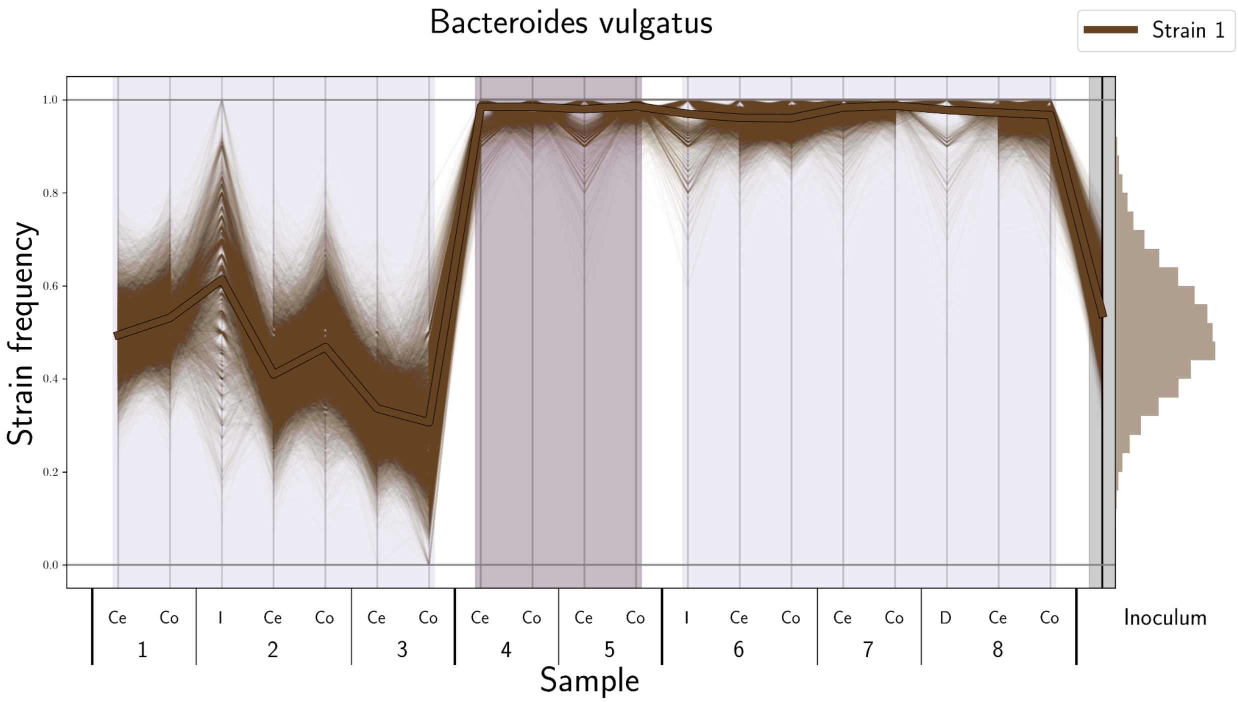

Supplementary Figure 14. Inferring strain frequency of *Bacteroides vulgatus* strains. Strain frequencies were inferred for *B. vulgatus* (and other species) by clustering loci into large groups of SNVs that display highly correlated allele frequencies (thin brown lines) across samples (indicated by labels on the x axis; "D" indicates duodenum, "I" indicates ileum, "Ce" indicates cecum, and "Co" indicates colon; numbers indicate mouse identity). The strain frequency was inferred to be the mean of the inferred clusters (thick brown line). In the case of *B. vulgatus*, one cluster was inferred, distinguishing two strains. The histogram to the right of the plot represents the distribution of allele frequencies of clustered SNVs in the inoculum.

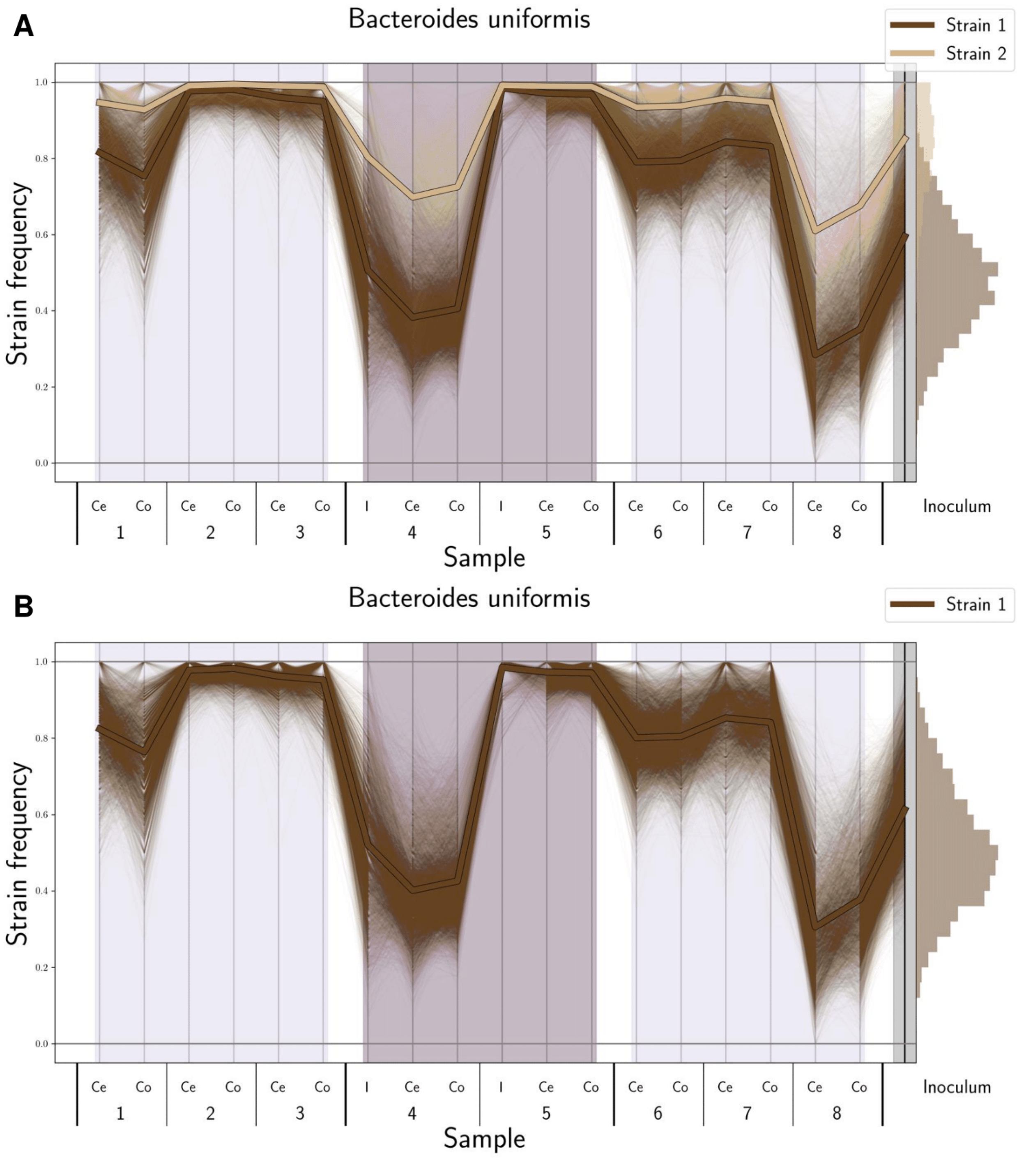

Supplementary Figure 15. Supervised strain frequency inference of *Bacteroides uniformis* strains. (A) *B. uniformis* SNVs were clustered into two groups, despite representing a single group of highly correlated SNVs. **(B)** The two clusters were merged, and SNVs that had a distance $d \leq 3.5$ with 25% of other SNVs in the cluster were retained (Methods).
